# Prevention of Periprosthetic Joint Infection in Total Hip and Knee Replacement: One European Consensus

**DOI:** 10.3390/jcm11020381

**Published:** 2022-01-13

**Authors:** Enrique Gómez-Barrena, Timothy Warren, Ian Walker, Neil Jain, Nanne Kort, François Loubignac, Simon Newman, Carsten Perka, Antonio Spinarelli, Michael R. Whitehouse, Luigi Zagra, Basilio J. De la Torre

**Affiliations:** 1 Department of Orthopaedic Surgery, Hospital La Paz, Autónoma University of Madrid, 28046 Madrid, Spain; 2 Triducive Ltd., Tunbridge Wells TN1 1NU, UK; tim.warren@triducive.com (T.W.); ian.walker@triducive.com (I.W.); 3 Department of Orthopaedic Surgery, Pennine Acute Hospitals NHS Trust, Manchester M8 5RB, UK; Neil.Jain@pat.nhs.uk; 4 CortoClinics, 5482 Schijndel, The Netherlands; nanne@cortoclinics.com; 5 CH Toulon, 83056 Toulon, France; francois.loubignac@ch-toulon.fr; 6 Grosvenor Orthopaedics, London W1G 9QW, UK; mrsimonnewman@gmail.com; 7 Department of Orthopaedic Surgery, Charité Hospital Universitätsmedizin, 10117 Berlin, Germany; carsten.perka@charite.de; 8 UOC Ortopedia e Traumatologia, Azienda Ospedaliero Universitaria Consorziale Policlinico di Bari, Università degli Studi di Bari “Aldo Moro”, 70121 Bari, Italy; antoniospinarelli@gmail.com; 9 Musculoskeletal Research Unit, Bristol Medical School, University of Bristol, Bristol BS8 1TH, UK; michael.whitehouse@bristol.ac.uk; 10 National Institute for Health Research Bristol Biomedical Research Centre, University Hospitals Bristol and Weston NHS Foundation Trust and University of Bristol, Bristol BS8 1TH, UK; 11 IRCCS Istituto Ortopedico Galeazzi, 20161 Milan, Italy; luigi.zagra@fastwebnet.it; 12 Department of Orthopaedic Surgery, Hospital Ramón y Cajal, University of Alcalá, 28034 Madrid, Spain; bjtorre@gmail.com

**Keywords:** prosthetic joint infection, total hip replacement, total knee replacement, perioperative PJI prevention

## Abstract

Periprosthetic joint infection (PJI) is a devastating complication in total hip and knee replacement. Its prevention is key to decrease the incidence and avoid some consequences that seriously impact patients and health systems. In view of the variety of recommendations and guidelines, we decided to conduct an expert, peer-reviewed European consensus analysis about the pre-, intra-, and postoperative prevention of PJI. A multinational group of practicing orthopedic experts developed a series of 47 consensus statements in 6 main groups of intervention, and a 2-stage Delphi approach was launched with a threshold for agreement at 75% and for very high agreement at more than 90%. A total of 306 orthopedic surgeon responses were gathered from 9 countries. Consensus was reached for 42/47 statements, 31/47 of which achieved a very high consensus. Many preoperative actions gathered strong consensus, although areas like the use of alcoholic chlorhexidine or the timing of hair removal did not attain strong consensus, despite available evidence. Intra- and postoperative actions showed more variability regarding incise drapes, skin suturing techniques, and wound follow-up. This study confirms an important consensus among orthopedic surgeons across Europe in many areas well known to contribute to the prevention of PJI; however, there are still grounds for improvement.

## 1. Introduction

Periprosthetic joint infection (PJI) occurs in around 1–2% of hip and knee primary arthroplasties. The number of cases is expected to rise due to an ageing population that has increasing expectations for mobility in older age [1,2]. The occurrence of PJI can lead to serious consequences, including chronic infection until loss of limb or amputation, with significant quality of life impairment [3]. If the infection invades the bloodstream and produces septicemia, even death can result [1,4]. It is reported that the five-year mortality associated with PJI is equal to that of oncology patients [5]. Revision surgery is more technically complex, requires more expensive prostheses, takes longer to perform, and is associated with greater blood loss and a higher rate of complications when compared to primary surgery. In the UK, revision surgery for infection is associated with an average length of hospital stay more than double that of aseptic cases and to an average cost more than three times that of an aseptic revision [6,7].

Along with individual patient burden, the occurrence of PJI is also a burden on healthcare systems. In the 5 years following surgery, the costs for patients undergoing total hip replacement revision surgery due to PJI are approximately five times greater than those for patients not requiring revision due to PJI [8], and in the first year, the costs for revised patient are over 13 times greater. 

PJI is caused by the introduction of microorganisms to the surgical site during surgery, through the bloodstream, by contiguous spread from another site, or due to recurrence from a previously infected joint [4]. There are many factors that can contribute to the risk of PJI, some of which are unmodifiable (e.g., age, history of previous joint infection, clotting disorders, prior transplant surgery), but many are modifiable (e.g., obesity, smoking, alcohol consumption, nutrition) [9,10]. The management of PJI may involve complex treatment strategies including surgical revision(s) and long-term targeted antimicrobial treatment. Various specialists with different approaches such as orthopedic and plastic surgeons, infectious disease physicians, and microbiologists are involved in the management of PJI. A multidisciplinary approach is therefore crucial for achieving optimal outcome [1].

Whilst international consensus exists (see https://icmphilly.com/, accessed 16 December 2021), there is currently no European specific consensus regarding best practice in surgical site infection (SSI) or PJI prevention, so regional specificities may not be considered. Thus, there is a need for convergence between guidelines and consensus at a European level. In order to minimize the significant burden associated with PJI, this consensus project was initiated to provide insights into the prevention of PJI in hip and knee surgery, informed by the opinions of orthopedic surgeons across Europe.

Due to the absence of European guidelines for SSIs/PJIs, the assumption was that variability exists in both practice and adherence to local, national, and international guidelines within Europe. We wished to evaluate and identify the key points that drive consensus with the objective of understanding the attitudes of surgeons towards the prevention of PJI associated with hip/knee surgery in Europe and define a consensus from a representative sample of respondents. In pursuing this objective, our group intends to understand attitudes and identify challenges within it, so that clear calls to action may be defined to support alignment regarding the optimal approach to minimize the risk of PJI.

## 2. Materials and Methods

A European expert-steering group met in November 2020 to review the current landscape and identify and agree about key domains in the hip/knee replacement care pathway regarding the prevention of PJI:

- Preventing and mitigating PJI according to risk factors
- Importance of skin preparation in preventing PJI
- The role of various options available to improve the pre-operative prevention of PJI
- What intra-operative actions could be implemented to improve the prevention of PJI
- What post-operative actions could be implemented to improve the prevention of PJI
- The role and suitability of the current guidelines

These domains were discussed to generate consensus statements for testing across a wider audience of orthopedic surgeons. Consensus statements (*n* = 47) were constructed to provide insight into surgical practice. The statements were then collated into a questionnaire, which was distributed electronically using Microsoft Forms to orthopedic surgeons in Europe. The recipients were identified either by the Steering Group or through their affiliated professional societies. The responses to the consensus statements were analyzed in line with Delphi methodology [11].

The respondent’s country was captured in the questionnaire to allow for the identification of any differences in practice at country level. Respondents were offered a 4-point Likert scale to rate their agreement with each statement, ranging across ‘strongly disagree’, ‘tend to disagree’, ‘tend to agree’, and ‘strongly agree’. Completed questionnaires were collated, and the individual scores for each statement were analyzed in order to produce an arithmetic agreement score for each (number of agreement level responses–number of disagreement level responses). The responses were broken down further by country to identify any variance in the respondent’s agreement scores. 

The steering group predefined agreement for consensus at 75%, a widely accepted threshold [12], and ‘very high’ agreement at ≥90%. Further rounds of questionnaire distribution were considered; however, due to the high levels of agreement for 42/47 statements, the group agreed that further rounds were not needed.

## 3. Results and Discussion

### 3.1. Respondent Demographics

**Figure 1:**
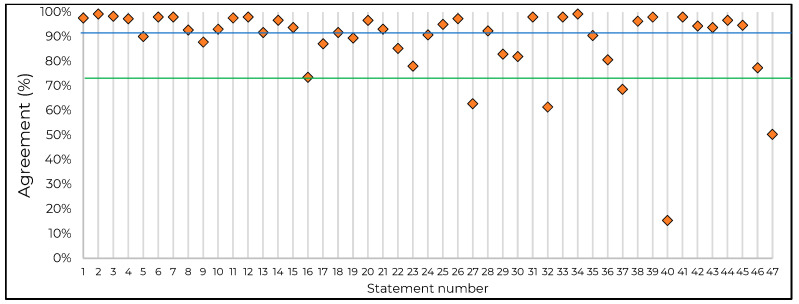
Consensus agreement by statement. Horizontal lines represent the thresholds for very high consensus (at 90%, in blue) and consensus (at 75%, in green). Orange diamonds represent the percentage of agreement per statement.

Completed questionnaires were returned by 306 orthopedic surgeons from various European countries (Germany, *n* = 101; France, *n* = 67; Italy, *n* = 65; UK, *n* = 34; Spain, *n* = 22; Austria, *n* = 6; Switzerland, *n* = 5; Belgium, *n* = 5; Netherlands, *n* = 1). The questionnaires were analyzed to define the total level of agreement with each of the 47 statements. Key domains grouping the statements are displayed in Table 1, Table 2, Table 3, Table 4, Table 5 and Table 6, which together with Figure 1, show the statements and corresponding agreement levels. 

### 3.2. Preventing and Mitigating PJI According to Risk Factors

The highest levels of consensus were reached for Domain A, and the results showed good alignment across countries. We found strong agreement that managing PJI requires a multidisciplinary approach (98%), and it has been suggested that multidisciplinary care can bring benefits at the unit, clinician, and patient level. One of the roles of a multidisciplinary team (MDT) is to bring collective knowledge to each patient and thereby agree a tailored approach to the management of risk factors for PJI. The MDT should include orthopedic surgeon, microbiologist, musculoskeletal radiologist, nutritionist, physiotherapist, and clinical nurse specialist members [13].

Prior to surgery, almost all respondents (97%) agreed individual risk factors should be assessed, and modifiable risk factors should be optimized (99%). Part of this process should involve patient education (98%) and agreement between stakeholders of the individual risk of PJI (90%). Poor glycemic control, obesity, malnutrition, and smoking (amongst other factors) are all associated with increased rates of PJI, whereas vitamin D replacement and *S. aureus* screening and treatment result in decreased rates [14].

Certainly, active patient participation in the pre-operative process should be encouraged, from reducing individual risk factors (98%), to cleansing at home prior to surgery (93%) using a chlorhexidine gluconate (CHG)-based soap or scrub solution (88%). Although contradictory or inconclusive results in the literature can be found about the efficacy of this measure [15,16], the presence of preoperative CHG in the skin increases if adequately applied at home [17] without adverse consequences, thus justifying the incorporation of this recommendation in guidelines [18]. The use of CHG-based soaps and scrubs is particularly recommended where the presence of *S. aureus* is detected/suspected [18]. These steps require active collaboration and should be supported by the sharing of appropriate information with the patient.

### 3.3. Importance of Skin Preparation in Preventing PJI

The respondents agreed that correct skin preparation is a key factor in preventing the occurrence of PJI. The first step in skin preparation is to ensure members of the surgical team receive appropriate training in PJI prevention (including skin preparation) and management (98%), and that appropriate patient education programs are in place (93%). Surgeons agree that the method of antiseptic use should be standardized (97%) with particular attention to the site of incision (93%). A visible antiseptic (i.e., colored for contrast against the skin) could help to ensure that all appropriate skin surfaces are treated (92%).

Although the use of antiseptics to reduce skin colonization is recommended (92%) as an important part of skin preparation (97%), the selection of the antiseptic agent is crucial, as antiseptics are not considered to be all equal (94%). Commonly used antiseptic agents include alcoholic chlorhexidine and iodine, both of which have been shown to reduce bacterial load on the skin prior to surgery. A 2019 network meta-analysis of the effectiveness of skin antiseptics in the prevention of SSI (including PJI) found that people who received chlorhexidine had a lower incidence of SSIs than people who received povidone–iodine, and the NICE evidence review committee recommended that alcohol preparations of chlorhexidine should be considered unless contraindicated, in which case alcohol povidone iodine should be used [19]. 

There was remarkable variation of agreement in response to statement 16 (Alcoholic chlorhexidine is significantly more protective than alcoholic povidone–iodine against both superficial incisional infections and deep incisional infections). Whilst responses from Germany, Italy, Spain, and the UK were in the range of 78–91% agreement, agreement from surgeons in France was significantly lower (49%). SF2H 2016 guidelines recommend the use of an alcoholic solution of chlorhexidine or povidone–iodine on healthy skin prior to invasive procedures, which may explain the almost 50–50 split in opinion [20]. WHO global guidelines 2016 recommend the use of an alcoholic solution of chlorhexidine, considered superior to povidone–iodine and reducing the incidence of SSI (these guidelines are not specific to orthopedic surgery) [21].

### 3.4. The Role of Various Options Available to Improve the Pre-Operative Prevention of PJI (22–26)

Whilst skin antisepsis is crucial to reduce the incidence of PJI, there are other adjunct methods that also contribute to this. Pre-operative patient processes should be designed to limit the risk of contamination during transfer to the operating room (OR, 85%). This may involve simple changes such as reducing the distance from the ward to the OR but may also include managing air pressure, temperature, humidity of the surgical environment, amongst other things [22]. If required, hair removal should be undertaken immediately prior to entering the OR (78%), using clippers rather than razors (91%). Surgical hand preparation should involve the use of alcohol-based solutions (95%). Prior to surgery, the patient should receive prophylactic antibiotic treatment (97%).

### 3.5. What Other Intra-Operative Action Could Be Implemented to Improve the Prevention of PJI (27–34)

There was almost universal agreement that traffic should be kept to a minimum in the OR (99%) and that the procedure time should be kept to a minimum to reduce the risk of PJI (98%). There was a more mixed response regarding the use of surgical drapes. Regarding statement 27 (incise drapes should not be mandatory in total joint replacement surgery, 63%), responses from France, Germany, and Italy did not achieve consensus (64%, 66%, and 66%, respectively) but were similar; the outliers were respondents from Spain who achieved consensus agreement (77%) and the UK (35%) where the majority of respondents disagreed with the statement. 

In the UK, the National Institute for Health and Care Excellence (NICE) states the following [18]: Do not use non-iodophor-impregnated incise drapes routinely for surgery, as they may increase the risk of SSI; if an incise drape is required, use an iodophor-impregnated drape, unless the patient has an iodine allergy.

This highlights a potential area of confusion, namely, that incise drapes (drapes that adhere to the skin and are cut through along with the underlying skin when an incision is made) may either be antibacterial-impregnated or -non-impregnated. If the respondent assumes that statement 27 applies to both types of incise drape, then, it may follow that their use should not be mandatory, whereas if the respondent assumes that the statement applies to antibacterial-impregnated incise drapes, then the response may differ. There may be some utility in investigating this discrepancy in future work. The steering group recognized that although incise drapes are increasingly used during surgery, there is conflicting evidence to support their use in terms of reducing PJI rates. Whilst some studies show that iodine-impregnated drapes are effective at preventing surgical wound infection with endogenous bacterial skin flora [23,24], a Cochrane systematic review showed that incise drapes may increase the rate of SSI due to more rapid colonization of the skin flora compared to bare skin [25]. Currently, the Society for Healthcare Epidemiology of America and the WHO recommended that neither antibacterial-impregnated or -non-impregnated incise drapes should be used routinely for the prevention of SSI [21,26]. On this basis, the use of incise drapes should not be mandatory. Where surgical drapes (of any kind) are used, these should be disposable to minimize the risk of transmission of pathogens to the surgical site (92%). 

Additionally, the use of any devices (particularly drains) that interact with the surgical site should be avoided (83%). Evidence suggests that the use of surgical drains has negligible impact on the risks of PJI when used for a short duration (≤24 h), but the risks of developing PJI increase significantly when the drain is in place for longer periods (≥48 h) [27]. There was also strong agreement about decreasing/minimizing hematoma to reduce the risk of infection (98%), and this may be achieved using tranexamic acid or hemostatic agents, the use of which may reduce the need for a surgical drain (82%). Based on these responses, tranexamic acid and hemostatic agents are recommended to routinely minimize hematoma and avoid the use of surgical drains where possible.

The suturing technique did not gather consensus regarding its impact on PJI rates (61%), which could be due to the definition and classification of superficial infection versus PJI. The suturing technique is important for superficial infection [28] but may not always be perceived as related to PJI. This could explain why the suturing technique was deemed less important based on these consensus results. 

### 3.6. What Other Post-Operative Action Could Be Implemented to Improve the Prevention of PJI (35–40)

There was a very high level of agreement on the fact that both surveillance and structured reporting of PJI are essential (96%, 98%, respectively). Surgical departments require that a robust system of surveillance is in place, appropriate to its patient demographic, resources, and hospital protocol.

The largely negative response to statement 40 was reassuring (an infection that appears after >30 days post-surgery is not considered to be a PJI, 15%). This statement was intended to be negative, and the very low levels of agreement suggest that the inverse is true, i.e., 85% of respondents agreed that PJI is not restricted to early wound infection (≤30 days). Therefore, infections appearing 30 days post-surgery may still be attributable to PJI.

Respondents agreed that prolonging antibiotic prophylaxis does not reduce the risk of PJI (91%), although there is some evidence of benefit in high-risk patients [29]. Both the WHO and the U.S. Centre for Disease Control (CDC) recommend against the use of antibiotics in the postoperative period, restricting the administration to just a single preoperative antibiotic [21,30]. Therefore, this guideline seems to be well aligned with practice.

Consensus agreement was not reached for statement 37, that the surgeon should review the patient operative wound periodically during the first month post-surgery (69%). Perhaps, it would be ideal if this were standard practice but, with time and resource pressures in healthcare systems, it may not be viable. This activity could be completed by an appropriately trained healthcare professional. Post-operative surveillance for PJI should be standardized based on best practice. Ianotti et al. [5] proposed a postoperative monitoring protocol for high-risk patients for up to 6 weeks post-surgery that focuses on several serological biomarkers, wound drainage, and multiple checks and management. 

### 3.7. The Role and Suitability of the Current Guidelines (41–47)

There is an identified need for focused evidence-based recommendations regarding the prevention of PJI in total joint surgery (98%). European specific consensus, similar to that produced as a result of the Second International Consensus Meeting (ICM) on orthopedic infections (see https://icmphilly.com/, accessed 16 December 2021), would be beneficial for surgical teams. If developed, such recommendations should be tailored to individual risk factors for PJI (94%), ideally with risk stratification protocols. 

The development of guidelines alone is not enough: effective implementation is the key to improving PJI. While not achieving consensus, the response to statement 47 did show that around 50% of surgeons in Europe believe that guidelines are not implemented in practice. Further analysis showed significant variation in response to statement 47 from individual countries, with Italy (74%) having the highest agreement levels. There is an opportunity to identify how and why this variance occurs in order to ensure that unwanted variations in practice are reduced. Any local (i.e., regional or hospital-specific) guidelines should be developed in line with national or European guidelines, and any areas of deviation should be evidentially justifiable based on local factors. Other sources of evidence should be used where there is a specific lack of level 1 or 2 evidence (95%).

Implementation of guidelines should use a considered approach involving evidence-based methodology. Beauchemin et al. [31] proposed a conceptual model from evidence innovation towards institutional, clinician, and patient assessment/education, and finally practice and poly deployment. More research is needed to clarify if other forms of fostering implementation, such as certification, could be useful. 

## 4. Limitations and Conclusions

As with all surveys, potential limitations of this study include the way in which the questions were worded and the order in which they were asked, and how respondents were approached. However, the questions were constructed by the steering group who also ratified the final form of the questionnaire before distribution. Many countries in Europe were not included (particularly from Eastern Europe), and this could be a limitation to spread the results, although many highly populated countries were present. There was a strong response from Germany (101/306), meaning that the overall results were heavily influenced by practice in Germany. That said, comparison of responses between Germany and other countries with a response above 20 individuals showed similar patterns of agreement with the statements. This consensus was focused specifically on clinical opinion, and patient experience was not captured. This input would be useful, particularly when discussing the expectations and preferences of patients.

PJI is a potentially life-threatening complication of hip and knee surgery associated with significant impacts on patient outcomes and increased healthcare resource utilization. The results of the consensus analysis provide a useful indication of the attitudes of orthopedic surgeons across Europe (including some variations between individual countries). Thus, the steering group was able to formulate a strong set of recommendations with the aim of minimizing the occurrence of PJI in the surgical setting. Broadly, optimizing the prevention of PJI involves both individual and systemwide activity, including education of healthcare professionals and patients regarding skin antisepsis, systematic use of antiseptics with the strongest evidence base, minimizing the use of devices that interact with the surgical site, and developing and implementing specific guidelines based on the strongest evidence and individualized for patient risk factors.

## 5. Recommendations

In hip and knee surgery, the following measures are recommended to minimize the risk of PJI:

1. Modifiable risk factors should be optimized prior to surgery
2. Patient education should involve skin cleaning techniques using a remnant antiseptic solution
3. Alcoholic chlorhexidine offers greater protection than alcoholic povidone–iodine against PJI
4. Alcohol-based solutions should be used in surgical hand preparation
5. A standardized approach to the use of antiseptics should be in place, with particular attention to the incision site
6. Antibiotic prophylaxis should be administered prior to surgery and not routinely prolonged
7. Traffic and number of personnel in the operating room should be kept to a minimum
8. Tranexamic acid and hemostatic agent use should be optimized to reduce the need for a surgical drain
9. Structured surveillance and reporting protocols for PJI should be in place
10. Specific guidelines for PJI should be developed and implemented; these should be tailored to individual patient risk factors
11. Guidelines based on level 1 or 2 evidence should be considered mandatory
12. Infections that appear 30 days post-surgery may still be considered to be PJI.

## Figures and Tables

**Table 1:** Consensus statements and agreement scores on Domain A: preventing and mitigating PJI according to risk factors.

No:	Statement	Score
1	The prevention and mitigation of periprosthetic joint infection (PJI) requires a multi-disciplinary approach	98%
2	Modifiable risk factors should always be optimized prior to any surgery	99%
3	Patient education is important when preventing and mitigating periprosthetic joint infection (PJI) according to risk factor	98%
4	The risk of surgery should be determined by the surgeon informing the patient about the individual risk factors	97%
5	The individual patient risk of periprosthetic joint infection (PJI) should be agreed via a shared decision-making process	90%
6	The patients should be empowered/encouraged to act and reduce their individual risk factors prior surgery (dietary, tobacco, alcohol)	98%
7	For high-risk patients, more focused information about preventing and mitigating periprosthetic joint infection (PJI) is required	98%
8	All elective-surgery patients should be requested to cleanse skin at home prior to surgery	93%
9	Cleansing at home should be done using a CHG-based soap or scrub solution	88%

**Table 2:** Consensus statements and agreement scores on Domain B: importance of skin preparation in preventing PJI.

No:	Statement	Score
10	Patient education about skin preparation is a vital part of effective periprosthetic joint infection (PJI) prevention	93%
11	Appropriate training of surgeons and nurses in periprosthetic joint infection (PJI) prevention helps reduce infection rates	98%
12	Appropriate training on optimal skin preparation techniques is effective in reducing periprosthetic joint infection (PJI) rates	98%
13	It is important to reduce any skin colonization prior to attending surgery by using an antiseptic solution	92%
14	The use of antiseptics is an important part of skin preparation	97%
15	Not all antiseptic solutions are equal	94%
16	Alcoholic chlorhexidine is significantly more protective than alcoholic povidone–iodine against both superficial incisional infections and deep incisional infections	75%
17	The remanence of the antiseptic used for skin preparation impacts the level of periprosthetic joint infection prevention	87%
18	Having visibility of where the antiseptic is applied impacts the level of periprosthetic joint infection (PJI) prevention	92%
19	The method of application of skin antiseptics is of high importance in maximizing their efficacy	90%
20	A standardized approach to applying and utilizing antiseptics improves the prevention of periprosthetic joint (PJI) infections	97%
21	Greater importance should be given to applying the antiseptic solution at the incision site	93%

**Table 3:** Consensus statements and agreement scores on Domain C: role of various options available to improve the pre-operative prevention of PJI.

No:	Statement	Score
22	There should be strict measures to limit the risk of contamination when the patient is transferred from the ward to the operating room	85%
23	Hair removal should be undertaken (if necessary) immediately prior to entering the operating room	78%
24	Hair removal is only recommended using clippers, not razors	91%
25	Surgical hand preparation should be achieved via the use of alcohol-based solutions to improve the prevention of periprosthetic joint infection	95%
26	Antibiotics should always be administered prophylactically to the patient prior to surgery	97%

**Table 4:** Consensus statements and agreement scores on Domain D: other intra-operative action that could be implemented to improve the prevention of PJI.

No:	Statement	Score
27	Incise drapes should not be mandatory in total joint replacement surgery	63%
28	Disposable drapes should be mandatory in total joint replacement surgery	92%
29	Devices or surgical drains that interact with the wound site should be avoided	83%
30	The use of tranexamic acid or hemostatic agents makes the use of surgical drains optional	82%
31	Decreasing hematoma (using tranexamic acid, hemostatic agents, or other) helps to reduce the risk of infection and avoid wound healing complications	98%
32	The choice of skin suturing technique strongly impacts the risk of periprosthetic joint infection (PJI)	61%
33	Shortening the surgical procedure duration reduces the risk of periprosthetic joint infection (PJI) in total joint replacement procedures	98%
34	Traffic should be kept minimal in the operating room during the time of surgery	99%

**Table 5:** Consensus statements and agreement scores on Domain E: what other post-operative action could be implemented to improve the prevention of PJI?

No:	Statement	Score
35	Prolonging surgical antibiotic prophylaxis does not reduce the risk of periprosthetic joint infection (PJI) in total joint replacement surgery	91%
36	Using advanced wound dressings reduces the risk of periprosthetic joint infection (PJI) in total joint replacement surgery	81%
37	The surgeon should review the patient operative wound periodically during the first month post-surgery	69%
38	Knowing and monitoring periprosthetic joint infection (PJI) rates proactively is essential to effective prevention	96%
39	Periprosthetic joint infection (PJI) occurrences should be reported in a structured way in the surgical department	98%
40	An infection that appears >30 days post-surgery is not considered to be a periprosthetic joint infection (PJI)	15%

**Table 6:** Consensus statements and agreement scores on Domain F: role and suitability of the current guidelines.

No:	Statement	Score
41	There is a need for focused recommendations about the prevention of periprosthetic joint infection (PJI) in total joint surgery	98%
42	Available guidelines need to be tailored to the individual risk factors of the patient	94%
43	There is a need to audit the compliance to recommendations followed	94%
44	Recommendations supported by Level 1 or 2 evidence should be considered mandatory	97%
45	When Level 1 or 2 evidence is lacking, other sources of evidence should be used to inform the prevention of periprosthetic joint infection (PJI) in total joint replacement surgery	95%
46	Local (hospital-based) recommendations should supersede international guidance in areas where evidence is lacking or divergent	77%
47	The majority of available recommendations are not implemented in practice	50%
